# Preparation of Peptoid Antifreeze Agents and Their Structure–Property Relationship

**DOI:** 10.3390/polym16070990

**Published:** 2024-04-04

**Authors:** Kang Yang, Di Liu, Lei Feng, Liugen Xu, Yangang Jiang, Xiran Shen, Amjad Ali, Jianwei Lu, Li Guo

**Affiliations:** School of Materials Science & Engineering, Jiangsu University, Zhenjiang 212013, China

**Keywords:** antifreeze agent, peptoid, ice recrystallization inhibition activity

## Abstract

The development of nontoxic and efficient antifreeze agents for organ cryopreservation is crucial. However, the research remains highly challenging. In this study, we designed and synthesized a series of peptoid oligomers using the solid-phase submonomer synthesis method by mimicking the amphiphilic structures of antifreeze proteins (AFPs). The obtained peptoid oligomers showed excellent antifreeze properties, reducing the ice crystal growth rate and inhibiting ice recrystallization. The effects of the hydrophobicity and sequence of the peptoid side chains were also studied to reveal the structure–property relationship. The prepared peptoid oligomers were detected as non-cytotoxic and considered to be useful in the biological field. We hope that the peptoid oligomers presented in this study can provide effective strategies for the design of biological cryoprotectants for organ preservation in the future.

## 1. Introduction

The formation and growth of ice crystals leads to impaired organ function during cryopreservation processes [1]. Worldwide, a large number of organs are wasted each year due to function loss caused by ice formed in cryopreservation [2,3]. Researchers have found that antifreeze agents can effectively reduce organ damage during cryopreservation by inhibiting the growth and recrystallization of ice crystals [4,5]. Currently, several antifreeze agents have been developed for cryopreservation, such as natural antifreeze proteins (AFPs) and synthetic antifreeze agents. AFPs can allow Antarctic organisms to survive in cold environment by lowering the freezing point of blood and inhibiting ice recrystallization [6,7,8]. However, their high extraction cost and serious biohazards limit the further applications of AFPs [9,10]. Synthetic antifreeze agents, such as dimethyl sulfoxide (DMSO), can effectively reduce the loss of cells in cryopreservation by regulating the osmotic pressure in cells. Nevertheless, DMSO is cytotoxic, which limits its application in the biological field [11]. Therefore, the development of biological antifreeze agents with a low price and excellent performance is urgently needed.

Researchers have tried to mimic the structures of AFPs and synthesize efficient antifreeze agents with high safety. Peptides composed of amino acids can mimic AFP structures easily and at the same time have good biocompatibility. Many studies have reported that peptides have broad potential applications in the field of antifreeze studies. Kim et al. studied the effects of some short peptides on ice crystal growth using molecular dynamics and found that the peptides can effectively inhibit the growth of ice crystals by binding to the ice crystal surface [12]. Zhang et al. designed and synthesized a series of peptide oligomers containing 12 amino acids as the anti-icing coating [13]. Gao et al. prepared a hydrophilic glycogenic peptide and applied it in the cryopreservation of human red blood cells [14]. However, peptides are unstable to enzymolysis, and their synthesis is complicated, which limits the wide use of peptides as antifreeze agents [15]. Peptoids are peptide mimics and have good biocompatibility, similar to that of peptides. The substitution of nitrogen instead of carbon on backbone makes peptoids have superior properties compared to peptides, such as better solubility and stability [16,17]. Studies on peptoids have been attracting more and more attention, and peptoids have been used in biomedicine [18,19], chemical catalysis [20] and biosensing [21]. More importantly, researchers have noticed that peptoids have the ability to change crystal structures. Zuckermann et al. synthesized a series of peptoid oligomers that can tune the morphology of CaCO_3_ crystals [22]. Kirshenbaum et al. found that peptoids can inhibit ice crystal growth and reduce the ice-melting temperature [23]. Kelland et al. reported the performance of peptoids as a kinetic hydrate inhibitor [24]. Inspired by these results, our group explored the properties of peptoids in inhibiting ice crystal formation and recrystallization and revealed the potential of peptoids as biological antifreeze agents [25,26]. However, the antifreeze properties of peptoids are still underexplored, and the relationship between peptoid structures and antifreeze activities has not been fully investigated.

In this study, we designed a peptoid side chain containing both hydrophilic and hydrophobic groups by mimicking the amphiphilic structure of an AFP. A series of peptoid oligomers containing amphiphilic groups were prepared using the solid-phase submonomer synthesis method, and their antifreeze properties were studied. The morphologies and growth rates of ice crystals were monitored through nanoliter osmometer experiments. The thermal hysteresis (TH) activities were analyzed by measuring the melting and growth temperatures of the ice crystals. The ice recrystallization inhibition (IRI) activities of the samples were characterized by measuring the mean largest grain sizes (MLGSs) of the ice crystals. Cytotoxicity was tested to ensure the safety of the peptoids as biological cryoprotectants. Significantly, the effects of changes in hydrophobic groups and sequence structures on the antifreeze properties of the peptoid oligomers were investigated. This study has instructional significance for the design of new biological cryoprotectants in the future.

## 2. Experimental Section

### 2.1. Materials

Dichloromethane, isopropanolamine, triethylamine, trimethylchlorosilane, anhydrous magnesium sulfate, rink amide resin (0.3~0.8 mmol/g), methanol, bromoacetic acid, dimethyldichlorosilance, 4-methylpiperidine, N-N-dimethylformamide (DMF) and N-N′-diisopropyl carbodiimide (DIC) were bought from Macklin (Shanghai, China). Methylamine aqueous, ethylamine, n-propylamine, isopropylamine, isobutylamine, ethanol, acetone, acetonitrile, triisopropylsilane (TIS) and trifluoroacetic acid (TFA) were bought from Aladdin (Shanghai, China). Deuterated chloroform and anhydrous ethanol were purchased from Sinopharm Chinese Reagent (Suzhou, China). Mouse fibroblasts (L929) were purchased from MeiSen Cell Biotechnology Ltd. (Hangzhou, China). Culture medium, trypsin and fetal bovine serum were bought from Cytiva (Logan, UT, USA). All solvents and reagents were used without further purification.

### 2.2. Synthesis of Monomers

Trimethylchlorosilane was reacted with isopropanolamine to form silane to protect the hydroxyl group. Firstly, 7.8 mL isopropanolamine and 24.4 mL triethylamine were added to 300 mL dichloromethane. The mixed solution was stirred in an ice bath for 10 min. Then, 16.1 mL trimethylchlorosilane was added slowly, and the obtained solution was stirred for 20 min. The ice bath was removed, and the solution was stirred at room temperature for another 16 h. The obtained solution was extracted with 150 mL of distilled water. The organic phase was dried with anhydrous magnesium sulfate, and then, the solvent was evaporated to obtain the product. The structure of the product was characterized via nuclear magnetic resonance spectroscopy (Bruker, 400 MHz, Billerica, MA, USA).

### 2.3. Synthesis of Peptoid Oligomers

The peptoid oligomers were synthesized using the solid-phase submonomer synthesis method [27]. The peptide synthesis tube was silanized for 30 min with a silicone solution (C_2_H_6_Cl_2_Si/DCM = 5/95, *v*/*v*), rinsed in dichloromethane and methanol, and then dried at room temperature. Then, 600 mg rink amide resin was swollen in 12 mL DMF and stirred via nitrogen bubbling for 10 min. The solution was drained with a vacuum to separate the swollen resin. In order to remove the protective Fmoc group, 6 mL 4-methylpiperidine solution (4-methylpiperidine/DMF = 1/4, *v*/*v*) was added and stirred for 2 min and 12 min, respectively, and drained. In total, 12 mL of DMF was added to wash the resin under nitrogen bubbles for at least 30 s, and then, the DMF was removed through vacuum filtration. The washing step was repeated 5 times. Afterward, 6 mL DMF solution of 0.6 M bromoacetic acid and 516 μL DIC was added and stirred for 20 min for acylation, and the solution was then removed through vacuum filtration. After a washing with DMF, 6 mL DMF solution of 1 M primary amine was added and reacted for 30 min. The required peptoid oligomers were obtained by repeating the acylation and replacement steps 6 times. After the reaction was completed, the resin was washed with DMF and methylene chloride through nitrogen bubbles, respectively. After drying, the resin was kept at −18 °C. The peptoid was obtained through cleavage from resin with 24 mL TFA solution (TFA/H_2_O/TIS = 95/2.5/2.5, *v*/*v*/*v*) for 1 h. The volatiles were evaporated by blowing nitrogen stream to obtain the crude peptoids. The structures of the peptoids were characterized via LC-MS (Thermo LXQ, Thermo Fisher Technology, Waltham, MA, USA) performed on a C18 column (AnPu, 4.6 mm × 250 mm, 5 μm). The solvent gradient (5–95%, acetonitrile/water, 0.1% TFA) was applied at a flow rate of 500 μL/min, and the analysis time was 45 min. The crude product was purified via reversed-phase high-performance liquid chromatography (RP-HPLC) (chromatographic column was 4.6 mm × 250 mm, 5 μm, Shimadzu, Kyoto, Japan) and freeze dried. The purified sample structures were characterized through a mass spectroscopy (MS) analysis. The purities of the peptoids after purification were characterized through an HPLC analysis.

### 2.4. Nanoliter Osmometer Experiments

A nanoliter osmometer experiment was conducted to characterize the morphologies and growth rates of ice crystals, as well as TH activities. The experiment employed a nanoliter osmometer (OsmoONE, INSTEC, Boulder, CO, USA) and a high-speed camera (PCO dimax S1, Excelitas PCO, Gottingen, Germany). The experimental environment was dehumidified before the test. Firstly, the peptoid oligomers dissolved in PBS buffer solution with different concentrations (1 mg/mL, 5 mg/mL and 10 mg/mL) were injected into a six-well plate coated with silicone oil, and the glass lid was quickly closed. The temperature was adjusted using the nano-rise osmometer to convert all the droplets into ice, and the temperature was raised slowly to melt the ice until the last ice crystal. The morphologies and growth processes of the ice crystals were observed using a microscope. The ice crystal growth data at different subcooling temperature ΔT (ΔT = 0.02~0.10 °C) were obtained by adjusting the temperature. The difference between the melting temperature and growth temperature of the ice crystal reflects the TH activity of the antifreeze agent. If the ice crystal does not exhibit a temperature difference between ice-growing and ice-melting temperatures, it means that the antifreeze agent does not have TH activity. The growth processes of the ice crystals were recorded using the high-speed camera, and ice crystals photos of different time periods were captured. The growth rates of the ice crystals were calculated using Image J calculation software (1.02.0005) (size growth of ice crystal divided by growth time). The ice crystal growth rate was obtained through a calculation of the average values of five experiments. In order to reduce experimental error, each group of samples was repeated at least three times.

### 2.5. Ice Recrystallization Inhibition Experiments

Ice recrystallization is a crucial factor leading to cell death after cryopreservation, and IRI activity is significant for biological antifreeze agents [28]. A Splata assay was used to characterize IRI activity [29]. The instrument was equipped with Linkam cryostage (C194, INSTEC, Boulder, CO, USA) and a Nikon polarization optical microscope (LV100ND, NIKON, Tokyo, Japan). In order to reduce the error, the experiment was carried out in a dry environment. First of all, the glass sheet was placed on a dry, low-temperature worktable and cooled to −60 °C at 20 °C/min and maintained for 3 min through the program. The solutions of peptoids dissolved in the PBS buffer solution with different concentrations (1 mg/mL, 5 mg/mL and 10 mg/mL) were dropped from a height of 1.5 m onto the center of the glass sheet to form a thin solid ice film. Then, the temperature was increased to −6 °C at a rate of 5 °C/min, and the morphologies and sizes of the ice crystals were observed with a microscope after the sample solutions were recrystallized for 30 min. Pictures of the ice crystals at different locations were taken using a high-speed camera in the next 20 min. The sizes of the 10 largest ice crystals in each picture were measured using Image J calculation software, and the average value was calculated. The mean largest grain size (MLGS) of the sample solutions was obtained using 10 photos of ice crystals at different locations. For each sample, the corresponding procedure was repeated at least three times. The relative percentage of the MLGS to PBS was used to characterize the IRI activity. 

### 2.6. Cytotoxicity Tests

Cytotoxicity testing is a crucial strategy used to test the safety of biological materials [30]. This experiment employed a low-speed centrifuge (SC-3610, Anhui Zhongke Zhongjia scientific Instrument Co., Ltd., Hefei, China), a cell incubator (CCL-170B-8, ESCO, Kuala Lumpur, Singapore) and a multifunctional microplate detector (Synergy H1, Bio-Tek, Winooski, VT, USA). In this test, the cytotoxicity of peptoid oligomers was detected using Cell Counting Kit-8 (CCK8, Finn Biological Co., Ltd., Wuhan, China). Mouse fibroblasts (L929) were cultured and subcultured to ensure that they met the experimental requirements. The old culture medium was removed from the cell dish, and 1 mL trypsin was added to digest the cells. A total of 2 mL of the new culture medium was added to the cell culture dish, and the cells were centrifuged to the bottom of the centrifuge tube. The culture medium was removed. An appropriate amount of new culture medium was added, and the centrifuge tube was shaken gently to disperse the cells evenly. A culture medium with a cell concentration of 60,000~80,000 cells per milliliter was inoculated into 96-well plates with 100 μL per well. It was cultured in a cell incubator (37 °C, 5% CO_2_) for 24 h. Peptoid oligomers were dissolved in the culture medium to obtain peptoid solutions with different concentrations (1 mg/mL, 5 mg/mL and 10 mg/mL). After filtration through a filter, a 100 μL peptoid solution was added to the corresponding well, and a blank control group containing only culture medium was added for 24 h. The old culture medium was removed, and 100 μL new culture medium was added, and 10 μL CCK-8 was added in a dark environment. Finally, the Optical Density (OD) at 450 nm was measured using a BioTek multifunctional microplate detector. The relative survival rate (σ) could be calculated using the following formula:

(1) $$\sigma =\frac{{OD}_{1}-{OD}_{0}}{{OD}_{2}-{OD}_{0}}\times 100\%$$

where OD_1_ was the average OD of the experimental group, OD_2_ was the average OD of the negative control group, and OD_0_ was the average OD of the blank control group. Cytotoxicity was characterized using the relative cell viability of the sample solution. The samples were considered to be non-cytotoxic when the relative viability of the cells was higher than 75%. Each sample was tested at least three times.

## 3. Results and Discussion

The two antifreeze characteristics of AFPs are TH and IRI activity. TH reflects the difference between the freezing and melting temperatures of ice crystals. IRI reflects the recrystallization and growth of ice crystals. For antifreeze agents used for cryopreservation, good IRI activity is significant, and TH activity is undesirable [31]. The amphiphilicity of AFPs has been believed to be critical to their IRI activities [32]. Threonines in AFPs can be used as ice-binding spots (IBSs) that affect the formation and growth of ice crystals. Both hydrophilic group hydroxyl and hydrophobic group methyl in the structure play crucial roles in binding with ice [32,33]. In imitating the structure of threonine, an amphiphilic functional group with both hydroxy and methyl called isopropyl alcohol group was designed as a peptoid side chain.

**Scheme 1:**
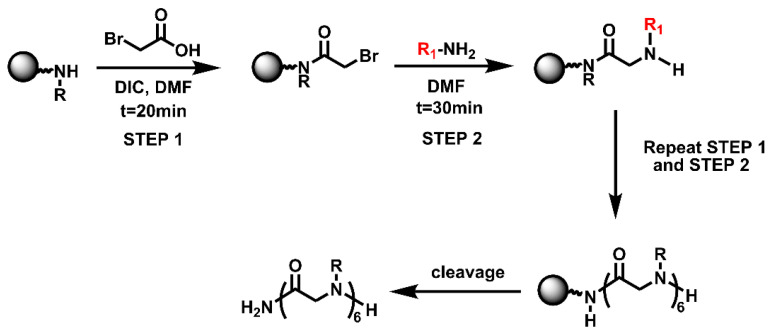
Synthesis route of peptoid oligomers.

The solid-phase submonomer synthesis method is the most effective method used to synthesize peptoid oligomers via repeated bromoacetic acylation and primary amine substitution [34]. The method can introduce different groups efficiently and orderly, which enriches the diversity of peptoid structures. In order to study the relationship between the structures of peptoid oligomers and their antifreeze activities, a series of isopropyl alcohol-containing peptoid oligomers (Scheme 1 and Table 1) were synthesized using the solid-phase submonomer synthesis method and purified via HPLC. The structure and purity of the oligomers were characterized via MS and HPLC to ensure that they met the experimental standards.

**Figure 1:**
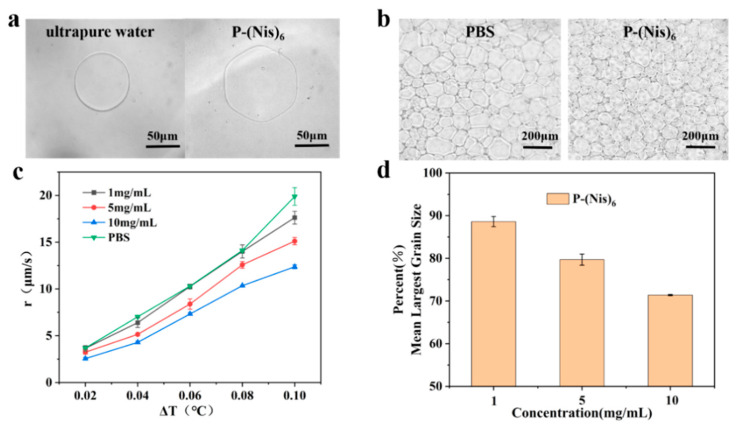
(**a**) Optical images of ice crystal morphology in ultrapure water and P-(Nis)_6_ solution with a concentration of 10 mg/mL at a ΔT of 0.08 °C; (**b**) ice crystal microscopic images of PBS buffer and P-(Nis)_6_ solution at 10 mg/mL after annealing at −6 °C for 30 min; (**c**) ice crystal growth rates of PBS buffer and P-(Nis)_6_ solution; (**d**) MLGSs in P-(Nis)_6_ solution as percentages of those in PBS buffer.

The peptoid oligomer P-(Nis)_6_ was designed and synthesized, and its antifreeze properties were characterized. As expected, P-(Nis)_6_ can interact with the ice crystal surface to change the ice morphology and inhibit ice growth. As shown in Figure 1a, P-(Nis)_6_ could transform the ice crystal shape from typical round to hexagonal and keep growing in a hexagon shape. The ice crystal growth rates in P-(Nis)_6_ solutions at different concentrations (1 mg/mL, 5 mg/mL and 10 mg/mL) were monitored. The results show that the ice crystal growth rate decreased with the increase in sample concentration. With the concentration of the P-(Nis)_6_ solution enhanced from 1 mg/mL to 10 mg/mL, the ice crystal growth rate decreased from 14.01 μm/s to 10.35 μm/s at a ΔT of 0.08 °C (Figure 1c). Compared with the ice in PBS buffer, the ice in the P-(Nis)_6_ solution grew at a similar rate when the peptoid concentration was low (1 mg/mL) and grew much slower when the peptoid concentration was higher (5 mg/mL and 10 mg/mL) at the same ΔT. In the experiment, no TH activity was observed for P-(Nis)_6_, which is desirable for the antifreeze agents used in cryopreservation [31]. At the same time, the MLGSs of the ice crystal after annealing at −6 °C for 30 min with different concentrations of P-(Nis)_6_ solutions were tested. The ice crystal MLGSs of the P-(Nis)_6_ solutions were lower than those of the PBS buffer, which proved that P-(Nis)_6_ has good IRI activity. With the increase in the sample concentration, P-(Nis)_6_ exhibited improved IRI activity (smaller MLGS). With the concentration of the sample solution increased from 1 mg/mL to 10 mg/mL, the MLGS of the ice crystal decreased from 147.8 μm to 121.8 μm (Figure 1b,d). These results indicate that the prepared P-(Nis)_6_ has an excellent ability to inhibit the growth and recrystallization of ice crystals.

**Figure 2:**
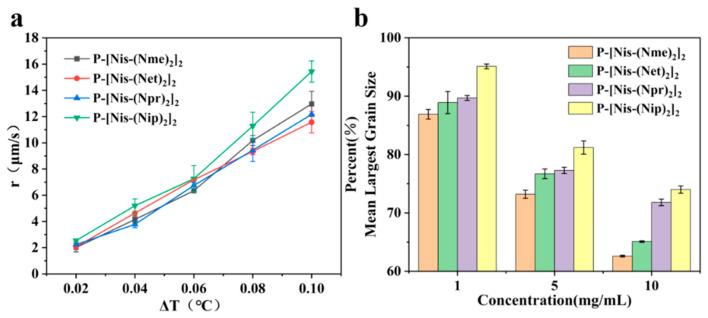
(**a**) Ice crystal growth rates in solutions of peptoid oligomers with different hydrophobic side chains at concentration of 10 mg/mL; (**b**) percentages of ice crystal MLGSs of peptoid oligomers with different hydrophobic side chains in PBS buffer.

Different hydrophobic groups were employed to further study the effect of the peptoid structure on antifreeze properties. Methyl, ethyl, propyl and isopropyl groups were, respectively, introduced into peptoid side chains to yield P-[Nis-(Nme)_2_]_2_, P-[Nis-(Net)_2_]_2_, P-[Nis-(Npr)_2_]_2_ and P-[Nis-(Nip)_2_]_2_. The peptoid oligomers containing hydrophobic side chains could also change the morphologies of ice crystals (Figure S4). Different concentrations of peptoid oligomers containing hydrophobic groups could reduce the growth rates of ice crystals, especially at higher concentrations (5 mg/mL and 10 mg/mL). In these peptoid solutions, the ice crystal growth rates decreased with an increase in concentration at the same ΔT. The results demonstrate that the ice crystal growth rates were comparative in the solution of peptoid oligomers when the hydrophobic side chain was methyl, ethyl or propyl (Figure 2a). For example, the ice crystal growth rates in the P-[Nis-(Nme)_2_]_2_, P-[Nis-(Net)_2_]_2_ and P-[Nis-(Npr)_2_]_2_ solutions at the concentration of 10 mg/mL and a ΔT of 0.02 °C were 2.01 μm/s, 2.07 μm/s and 2.24 μm/s, respectively. However, the peptoid with isopropyl as a hydrophobic side chain, i.e., P-[Nis-(Nip)_2_]_2_, exhibited a relatively poor ability to inhibit ice crystal growth due to the large steric hindrance of isopropyl, which could affect the interactions between peptoid molecules and the ice crystal surface. All peptoid oligomers containing different hydrophobic groups had no TH activity. The IRI activity was also characterized. Basically, all these amphiphilic peptoids can inhibit ice crystal recrystallization and showed smaller MLGSs than the PBS buffer (Figure 2b). With the increase in hydrophobic side chain length, i.e., increase in hydrophobicity, the peptoid showed decreased IRI activity, which indicated the importance of the balance between the hydrophilicity and hydrophobicity of the peptoids. For example, the percentage of MLGS was 62.6% in P-[Nis-(Nme)_2_]_2_ solution and 71.8% in P-[Nis-(Npr)_2_]_2_ solution when the concentration was 10 mg/mL. The propyl and isopropyl groups have similar hydrophobicity values, but the peptoid with propyl side chains showed better IRI activity. This can be attributed to the steric hindrance of the isopropyl group affecting the interactions between the ice surface and peptoids. Overall, the peptoids with isopropyl hydrophobic side chains had less antifreeze activities than those with propyl side chains. Therefore, side chains with large steric hindrance groups should be avoided in peptoid antifreeze agents. 

**Figure 3:**
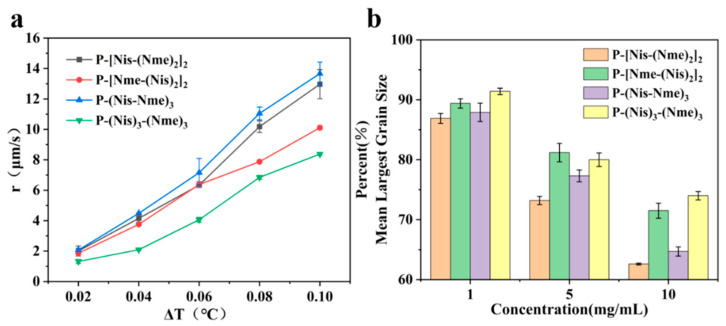
(**a**) Ice crystal growth rates of peptoid oligomers with different sequences at concentration of 10 mg/mL; (**b**) percentages of ice crystal MLGSs of peptoid oligomers with different sequences in PBS buffer.

The peptoid oligomers with the methyl hydrophobic group exhibited the best antifreeze ability in all samples. Different sequences of peptoid oligomers containing isopropyl alcohol group and methyl group were synthesized to further investigate the effect of the sequence structure on the antifreeze properties. All the prepared peptoid sequences showed the ability to inhibit ice crystal growth and recrystallization (Figure 3). The results indicate that block sequence P-(Nis)_3_-(Nme)_3_ exhibited the most excellent ability to inhibit ice crystal growth, which is consistent with the results of previous studies (Figure 3a) [26]. A high concentration is favorable to reduce the ice crystal growth rate. For P-(Nis)_3_-(Nme)_3_ solution at a ΔT of 0.08 °C, the ice crystals growth rate changed from 11.23 μm/s to 6.85 μm/s when the concentration was increased from 1 mg/mL to 10 mg/mL (Figure S5). None of the synthesized peptoid oligomers with different sequences showed TH activity. The ice crystal MLGSs in peptoid oligomer solutions also decreased with the increase in concentration (Figure 3b). For P-(Nis-Nme)_3_ solution, the percentage of MLGS decreased from 87.9% to 64.7% when the concentration was raised from 1 mg/mL to 10 mg/mL. Among all sequences of peptoid oligomers, P-[Nis-(Nme)_2_]_2_ possessed the best IRI activity. This is because the sequence of isopropyl alcohol and methyl groups in P-[Nis-(Nme)_2_]_2_ is the same as the sequence of threonine and alanine in antifreeze glycoproteins [35]. The MLGSs of ice crystals in P-[Nis-(Nme)_2_]_2_ solution accounted for only 62.6% of the PBS buffer solution at the concentration of 10 mg/mL (Figure 3b). The IRI activities of P-(Nis-Nme)_3_ and P-[Nis-(Nme)_2_]_2_ were similar. P-[Nme-(Nis)_2_]_2_ and P-(Nis)_3_-(Nme)_3_ demonstrated a relatively poor ability to inhibit ice crystal recrystallization. At the concentration of 10 mg/mL, the ice crystal MLGSs in the P-[Nme-(Nis)_2_]_2_ and P-(Nis)_3_-(Nme)_3_ solutions were 71.5% and 74.0% of the PBS buffer, respectively. However, they showed a good ability to inhibit the growth of ice crystals. The peptoid oligomers with different sequence structures exhibited different antifreeze activities. Peptoid oligomers of the same structure could have different effects on inhibiting ice crystal growth and recrystallization. Therefore, the effects of the peptoid structure on the antifreeze properties are important but complicated. 

**Figure 4:**
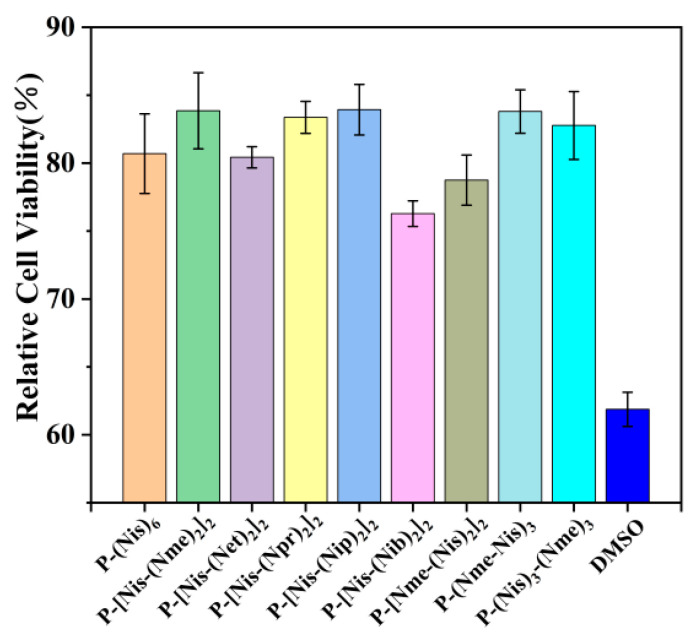
The relative cell viability of peptoids and DMSO at the concentration of 10 mg/mL.

To further verify the potential of peptoid oligomers as biological cryoprotectants, cytotoxicity tests were performed. DMSO, as a common cryoprotectant in practical applications, was used to compare with peptoids in a cytotoxicity test. Generally speaking, samples with a cell survival rate of less than 75% are considered to be cytotoxic [36]. The results show that the relative cell viability of all peptoid oligomer solutions was greater than 75% at a concentration of 10 mg/mL, which is considered to be non-cytotoxic (Figure 4). The relative cell viability of DMSO was only 61.9%, confirming the presence of cytotoxicity. It can be concluded that the peptoid antifreeze agents prepared here have comparative or better antifreeze properties than DMSO but safer (Figures S6, S9 and S10). The results of this study undoubtedly confirm the potential of peptoids as antifreeze agents in cryopreservation. 

## 4. Conclusions

In summary, we designed a peptoid with an amphiphilic functional group as a side chain by imitating the structure of an AFP, which has hydrophilic and hydrophobic groups simultaneously. Furthermore, a series of peptoid oligomers with different hydrophobicity values and sequences were synthesized to study the relationship between structure and antifreeze properties. The prepared peptoid oligomers could change the morphologies of ice crystals from round to hexagonal by interacting with the ice crystals. The peptoids had the ability to inhibit ice crystal growth and recrystallization. All peptoid oligomers proved to have no TH activity. The antifreeze activities of peptoid oligomers improved with an increase in concentration. The results show that the hydrophobic group activity had little effects on the ability of the peptoid to inhibit the growth of the ice crystals. With an increase in hydrophobicity, however, the IRI activity of the peptoid oligomers decreased. Groups with a high steric hindrance had negative effects on the antifreeze properties of the peptoid oligomers and should be avoided. Among all the sequences, P-(Nis)_3_-(Nme)_3_ displayed the best ability to inhibit ice crystal growth, and P-[Nis-(Nme)_2_]_2_ possessed the best IRI activity. Cytotoxicity tests were carried out to prove the safety of the peptoids. With good antifreeze properties and without being cytotoxic, the peptoids presented here have great potential as antifreeze agents in cryopreservation. The relationship between the structures and antifreeze performances of the peptoids was studied, which is helpful in the development and application of biological antifreeze agents in the future.

## Figures and Tables

**Table 1:** Structures of the prepared peptoid oligomers.

Sample Name	Structure
P-(Nis)_6_	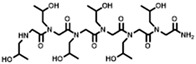
P-[Nis-(Nme)_2_]_2_	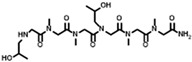
P-[Nis-(Net)_2_]_2_	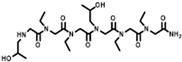
P-[Nis-(Npr)_2_]_2_	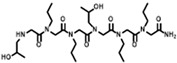
P-[Nis-(Nip)_2_]_2_	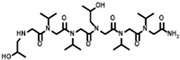
P-[Nme-(Nis)_2_]_2_	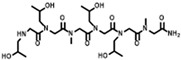
P-(Nis-Nme)_3_	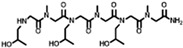
P-(Nis)_3_-(Nme)_3_	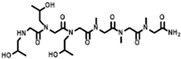

## Data Availability

Data are contained within the article and Supplementary Materials.

## Supplementary Materials

The following supporting information can be downloaded at: https://www.mdpi.com/article/10.3390/polym16070990/s1: Scheme S1: Synthesis route of submonomer 3-trimethylsilanoxy-propylamine. Figure S1: ^1^H NMR spectrum of 3-trimethylsilanoxy-propylamine in CDCl_3_; Figure S2: MS spectra of peptoid oligomers. (a) P-(Nis)_6_; (b) P-[Nis-(Nme)_2_]_2_; (c) P-[Nis-(Net)_2_]_2_; (d) P-[Nis-(Npr)_2_]_2_; (e) P-[Nis-(Nip)_2_]_2_; (f) P-[Nme-(Nis)_2_]_2_; (g) P-(Nis-Nme)_3_; (h) P-(Nis-Nme)_3_; Figure S3: HPLC traces after purification of peptoid oligomers. (a) P-(Nis)_6_; (b) P-[Nis-(Nme)_2_]_2_; (c) P-[Nis-(Net)_2_]_2_; (d) P-[Nis-(Npr)_2_]_2_; (e) P-[Nis-(Nip)_2_]_2_; (f) P-[Nme-(Nis)_2_]_2_; (g) P-(Nis-Nme)_3_; (h) P-(Nis-Nme)_3_; Figure S4: Optical images of ice crystal morphology in peptoid solutions and ultrapure water at the concentration of 10 mg/mL (ΔT = 0.08 °C). (a) ultrapure water; (b) P-(Nis)_6_; (c) P-[Nis-(Nme)_2_]_2_; (d) P-[Nis-(Net)_2_]_2_; (e) P-[Nis-(Npr)_2_]_2_; (f) P-[Nis-(Nip)_2_]_2_; (g) P-[Nme-(Nis)_2_]_2_; (h) P-(Nis-Nme)_3_; (i) P-(Nis-Nme)_3_; Figure S5: Comparison of ice crystal growth rates between peptoid oligomers solutions and PBS buffer. (a) P-(Nis)_6_; (b) P-[Nis-(Nme)_2_]_2_; (c) P-[Nis-(Net)_2_]_2_; (d) P-[Nis-(Npr)_2_]_2_; (e) P-[Nis-(Nip)_2_]_2_; (f) P-[Nme-(Nis)_2_]_2_; (g) P-(Nis-Nme)_3_; (h) P-(Nis-Nme)_3_; Figure S6: The ice crystal growth rates of all peptoid and DMSO solutions at the concentration of 10 mg/mL.; Figure S7: Micrographs of ice crystals grown in 10 mg/mL solution of peptoid solutions and PBS buffer. (a) PBS; (b) P-(Nis)_6_; (c) P-[Nis-(Nme)_2_]_2_; (d) P-[Nis-(Net)_2_]_2_; (e) P-[Nis-(Npr)_2_]_2_; (f) P-[Nis-(Nip)_2_]_2_; (g) P-[Nme-(Nis)_2_]_2_; (h) P-(Nis-Nme)_3_; (i) P-(Nis-Nme)_3_; Figure S8: The MLGS of P-(Nis)_6_ solution were compared with PBS buffer; Figure S9: Percentage of MLGS of ice crystals in PBS buffer after peptoids and DMSO solutions annealing at −6 °C for 30 min; Figure S10: The relative cell viability of peptoids and DMSO solutions at different concentrations.
