# Supplementary material for: Preparation of Peptoid Antifreeze Agents and Their Structure–Property Relationship
Source: Polymers (Basel). 2024 Apr 4;16(7):990. doi: 10.3390/polym16070990 (PMC11013998; doi:10.3390/polym16070990)
Supplement: Supplementary file 1 [file polymers-16-00990-s001.zip › polymers-2916374-supplementary.pdf]

Supplementary Information

Preparation of Peptoid Antifreeze Agents and Their Structure-Property Relationship Study

Kang Yang, Di Liu, Lei Feng, Liugen Xu, Yangang Jiang, Xiran Shen, Amjad Ali,

Jianwei Lu*, Li Guo*

School of Materials Science & Engineering, Jiangsu University,

Zhenjiang 212013, China

<u>Table of contents</u>

1. Monomer synthesis and characterization 2
2. MS spectra of peptoid oligomers…………………………………………………3
3. HPLC spectra of peptoid oligomers……………………………………………...4
4. The morphologies of ice crystals..5
5. The growth rates of ice crystals…………………………………….......................6
6. Ice recrystallization inhibition activities of peptoid oligomers….…………………..7
7. Cytotoxicity test results……………………………………......………....…...8

1. Monomer synthesis and characterization

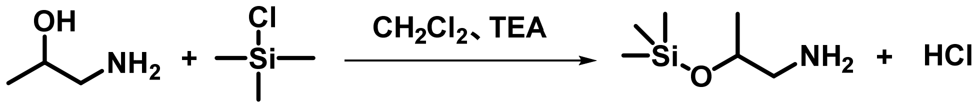

Scheme S1 Synthesis route of submonomer 3-trimethylsilanoxy-propylamine.

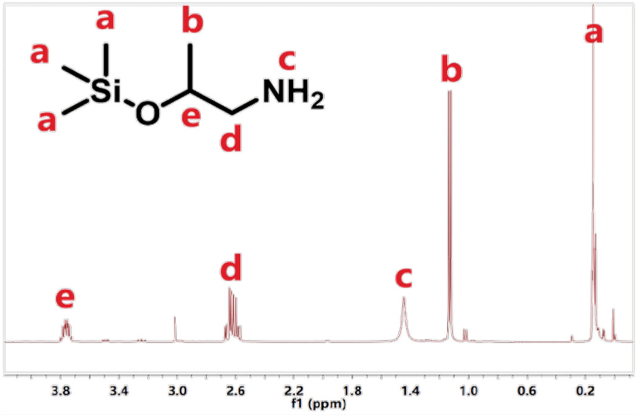

Fig. S1 ^1H NMR spectrum of 3-trimethylsilanoxy-propylamine in $CDCl_3$

2. MS spectra of peptoid oligomers

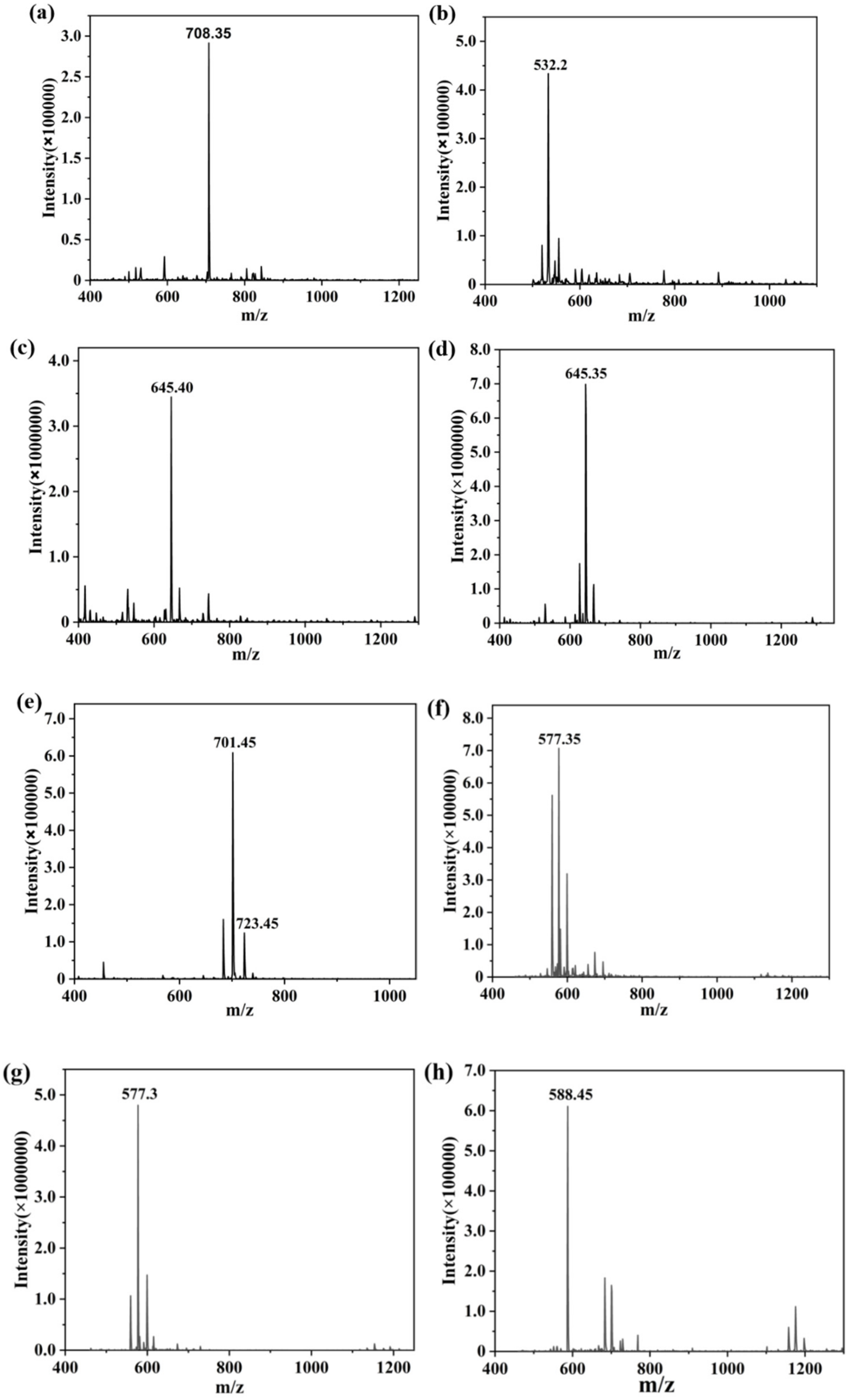

Fig. S2 MS spectra of peptoid oligomers. (a) P-$(Nis)_6$; (b) P-$[Nis\text{-}(Nme)_2]_2$; (c) P-$[Nis\text{-}(Net)_2]_2$; (d) P-$[Nis\text{-}(Npr)_2]_2$; (e) P-$[Nis\text{-}(Nip)_2]_2$; (f) P-$[Nme\text{-}(Nis)_2]_2$; (g) P-$(Nis\text{-}Nme)_3$; (h) P-$(Nis\text{-}Nme)_3$

3. HPLC spectra of peptoid oligomers

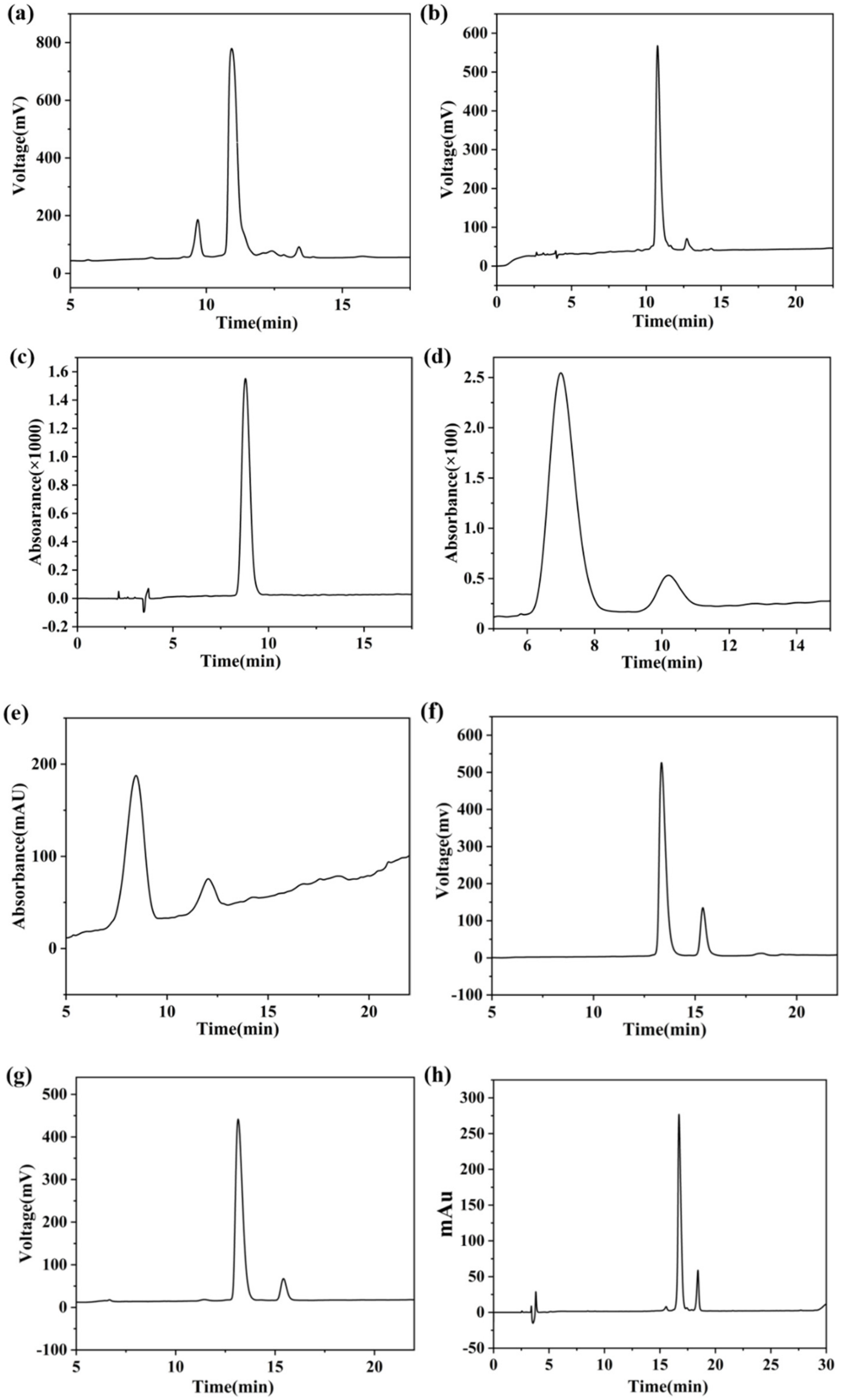

Fig. S3 HPLC traces after purification of peptoid oligomers. (a) P-$(Nis)_6$; (b) P-$[Nis\text{-}(Nme)_2]_2$; (c) P-$[Nis\text{-}(Net)_2]_2$; (d) P-$[Nis\text{-}(Npr)_2]_2$; (e) P-$[Nis\text{-}(Nip)_2]_2$; (f) P-$[Nme\text{-}(Nis)_2]_2$; (g) P-$(Nis\text{-}Nme)_3$; (h) P-$(Nis\text{-}Nme)_3$

4. The morphologies of ice crystals

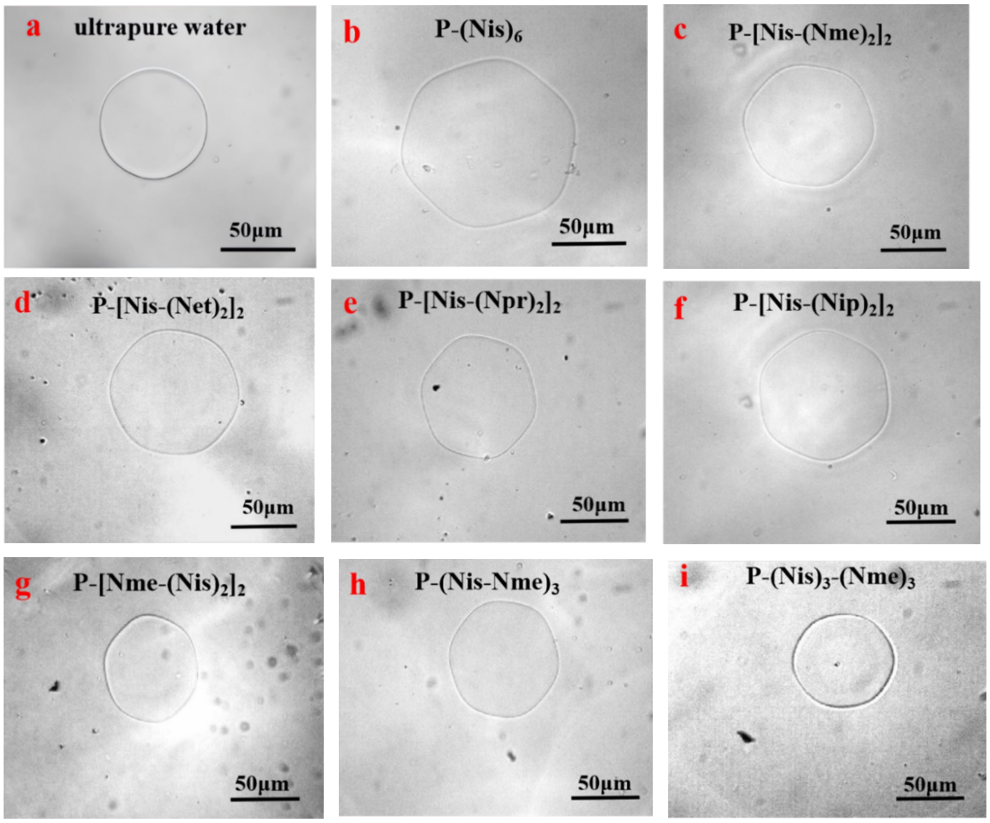

Fig. S4 Optical images of ice crystal morphology in peptoid solutions and ultrapure water at the concentration of 10 mg/mL (ΔT = 0.08 °C). (a) ultrapure water; (b) P-$(Nis)_6$; (c) P-$[Nis\text{-}(Nme)_2]_2$; (d) P-$[Nis\text{-}(Net)_2]_2$; (e) P-$[Nis\text{-}(Npr)_2]_2$; (f) P-$[Nis\text{-}(Nip)_2]_2$; (g) P-$[Nme\text{-}(Nis)_2]_2$; (h) P-$(Nis\text{-}Nme)_3$; (i) P-$(Nis\text{-}Nme)_3$.

5. The growth rates of ice crystals

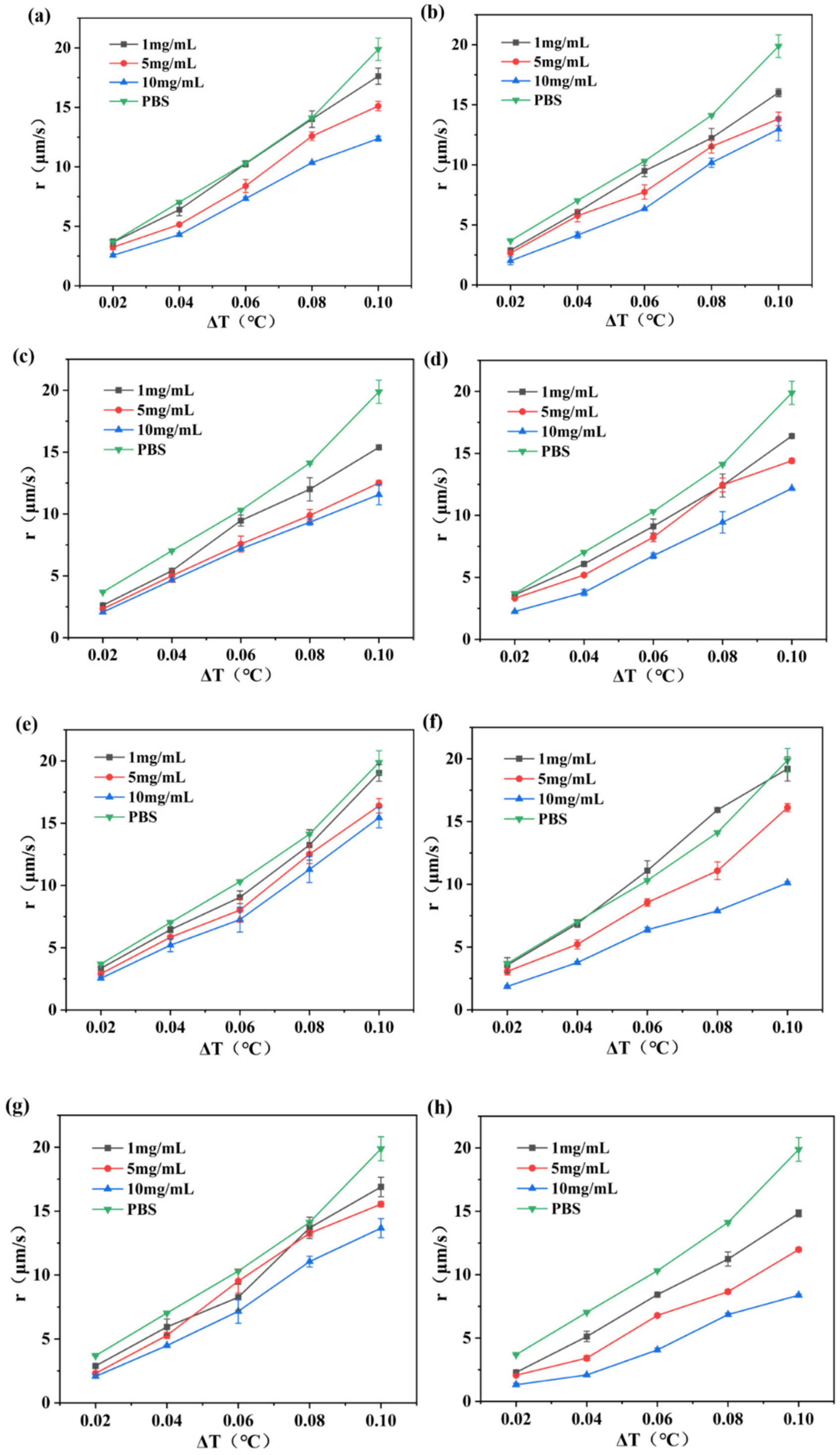

Fig. S5 Comparison of ice crystal growth rates between peptoid oligomers solutions and PBS buffer. (a) P-$(Nis)_6$; (b) P-$[Nis\text{-}(Nme)_2]_2$; (c) P-$[Nis\text{-}(Net)_2]_2$; (d) P-$[Nis\text{-}(Npr)_2]_2$; (e) P-$[Nis\text{-}(Nip)_2]_2$; (f) P-$[Nme\text{-}(Nis)_2]_2$; (g) P-$(Nis\text{-}Nme)_3$; (h) P-$(Nis\text{-}Nme)_3$

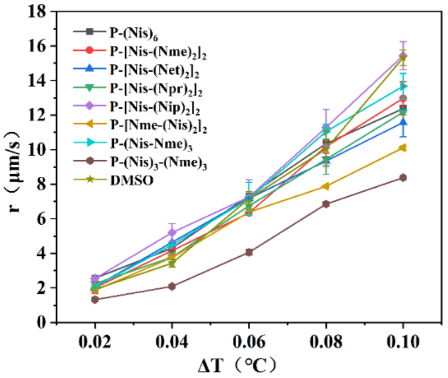

Fig. S6 The ice crystal growth rates of all peptoid and DMSO solutions at the concentration of 10 mg/mL.

6. Ice recrystallization inhibition activities of peptoid oligomers

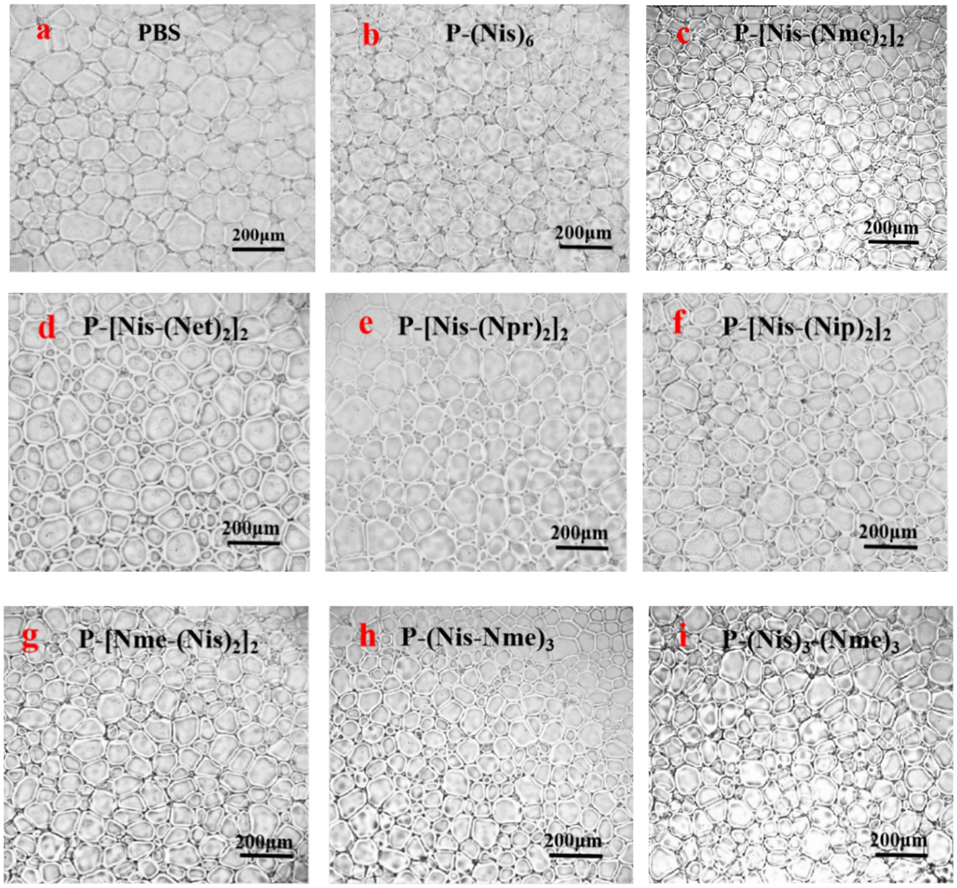

Fig. S7 Micrographs of ice crystals grown in 10 mg/mL solution of peptoid solutions and PBS buffer. (a) PBS; (b) P-$(Nis)_6$; (c) P-$[Nis\text{-}(Nme)_2]_2$; (d) P-$[Nis\text{-}(Net)_2]_2$; (e) P-$[Nis\text{-}(Npr)_2]_2$; (f) P-$[Nis\text{-}(Nip)_2]_2$; (g) P-$[Nme\text{-}(Nis)_2]_2$; (h) P-$(Nis\text{-}Nme)_3$; (i) P-$(Nis\text{-}Nme)_3$.

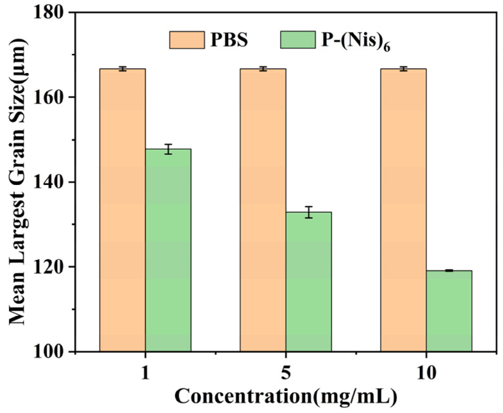

Fig. S8 The MLGS of P-(Nis)$_6$ solution were compared with PBS buffer.

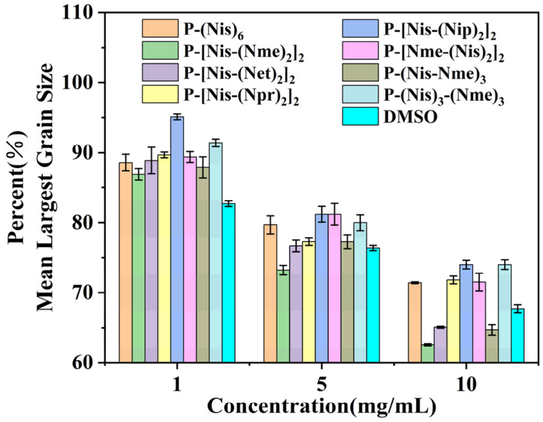

Fig. S9 Percentage of MLGS of ice crystals in PBS buffer after peptoids and DMSO solutions annealing at -6 ℃ for 30 min.

7. Cytotoxicity test results

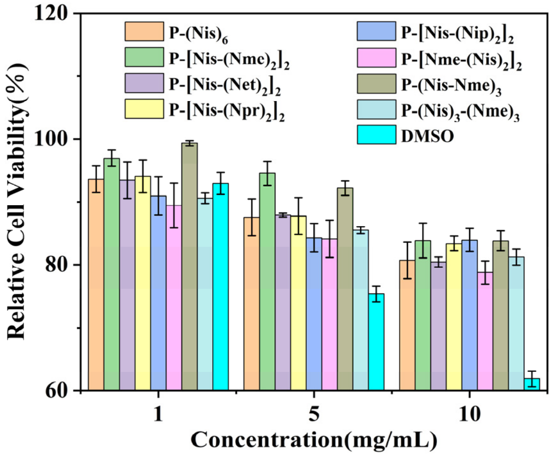

Fig. S10 The relative cell viability of peptoids and DMSO solutions at different concentrations.
